# Relationship between Mothers’ Nutritional Knowledge in Childcare Practices and the Growth of Children Living in Impoverished Rural Communities

**Published:** 2014-06

**Authors:** Mahama Saaka

**Affiliations:** University for Development Studies, School of Medicine and Health Sciences, PO Box 1883, Tamale, Ghana

**Keywords:** Childcare knowledge index, Child nutrition, Interaction effect, Socioeconomic status, Ghana

## Abstract

This study assessed the relationship between maternal nutritional knowledge in childcare practices and growth of children living in impoverished rural communities. This was an analytical cross-sectional study which covered a random sample of 991 children aged 0-36 month(s). Multivariate analysis showed that, after adjusting for potential confounders, there was a significant positive association between the childcare knowledge index and mean HAZ (β=0.10, p=0.005) but was not associated with mean WHZ. The strength of association increased among women of high socioeconomic status (β=0.15, p=0.014) but there was no significant association among women of low socioeconomic status. Increase in maternal childcare knowledge may contribute significantly to child's nutritional status in Ghana if there is concurrent improvement in socioeconomic circumstances of women living in deprived rural communities.

## INTRODUCTION

The prevalence of chronic malnutrition among under-five children remains persistently high in Ghana. For example, in the Northern Region of Ghana, 32.5% of children below five years are stunted, 12.9% wasted, and 21.8% underweight (1). Inadequate childcare practices are fundamental to addressing malnutrition among children. Poor maternal education (formal and informal) has been identified as a major constraint to good childcare practices in Ghana (2). A well-resourced, targeted and coordinated nutrition education can improve maternal nutritional knowledge, healthcare-seeking behaviours, and practices significantly. Consequently, a number of health-related non-governmental organizations, including Catholic Relief Services (CRS), Adventist Development and Relief Agency (ADRA), World Vision International (WVI), and the Ghana Health Service (GHS) have been promoting proper childcare practices, including appropriate infant-feeding practices and management of childhood illnesses, such as diarrhoea. Health and nutrition messages are usually targeted to mothers, most of whom have not received formal education. These women usually patronize health services at antenatal clinics and child welfare centres (CWC). Additionally, patronage of preventive health services provides an opportunity to improve care practices through both preventive healthcare (immunization, antenatal care for the mother, etc.) as well as management of childhood morbidity. Effective utilization of knowledge and skills gained from health and nutrition education is, therefore, expected to improve the health and nutritional status of children through improved knowledge and care practices. However, there are limited data on the impact of nutrition education, especially in women who have not received formal education. Care behaviour choices are mediated by knowledge as well as by resource availability. Practices and behaviours of individuals are influenced by knowledge, awareness and skill levels. Even in households with similar levels of access to disposable income and resource, there is a wide variation in nutritional outcomes of children (3), which tends to suggest that factors other than resources are responsible for nutritional status of children. Adequate childcare is an underlying factor for optimal growth. Caregiving behaviours that provide conducive environment within which children are raised are central to nutritional outcomes of children, and policy attention to them has been recommended by the International Conference on Nutrition (4).

The fundamental role of care to child nutrition has been well-established since 1990 through UNICEF Model of Care. To provide care adequately, caregivers require education (both formal and informal), time, and support (e.g. control of resources). In the Ghanaian context, it remains unclear what the relationship is between the nutritional knowledge of non-literate mothers and nutritional status of their children. One would expect that mothers’ knowledge of child nutrition and childcare practices would have a significant effect on their children's nutritional status. However, there are conflicting study results on this.

Whereas some studies have reported that maternal nutritional knowledge is positively associated with the nutritional status of children (5-7), others have also shown that adequate knowledge *per se* is not always translated into appropriate actions (8-10). Understanding the factors that determine the translation of adequate child health and nutritional knowledge into appropriate action in impoverished environment might help design more effective interventions against malnutrition. It remains unclear whether giving mothers adequate knowledge on proper childcare practices has an independent impact on child growth. This study, therefore, investigated the relationship between mother's knowledge level in childcare practices and nutritional status of preschool children living in impoverished rural communities of Northern Ghana.

### Statement of the problem

In Ghana, health and nutrition education is a common intervention targeted to mothers, most of whom have not received formal education. However, inadequate empirical evidence exists on the relationship between nutritional knowledge of non-literate mothers and nutritional status of their children. Although maternal education is an important determinant of nutritional status of children, it remains unclear whether mother's practical knowledge about nutrition has an independent effect on child growth. Furthermore, the factors that influence translation of acquired knowledge into practice are not well-understood. It is this knowledge gap that this study attempts to address.

### Research questions

- What is the level of maternal knowledge in recommended childcare practices in the study population?
- Does household socioeconomic status influence mother's ability to translate acquired knowledge into practice in order to improve the nutritional status of children in impoverished rural communities of Ghana?
- Is there a direct link between child growth and a composite childcare knowledge index?

### Study objectives

The main aim of the study was to assess the relationship between maternal nutritional knowledge in childcare practices and child growth among children aged 0-36 month(s) in Northern Ghana.

The specific objectives of the study were to: (i) assess mothers’ knowledge level in recommended childcare practices relating to nutrition; (ii) investigate whether translation of acquired health and nutritional knowledge into practice is dependent on the socioeconomic status of the household in impoverished rural communities; (iii) assess the nutritional status of infants and young children aged 0-36 month(s); and (iv) build a composite childcare knowledge index and investigate its relationship with nutritional indicators in children.

### Significance of the study

In order for policy-makers to better promote change and improve children's wellbeing in impoverished communities, it is necessary to provide more insight into the relationship between maternal knowledge and child health outcomes. The circumstances under which acquired knowledge is put into practice are all important in deciding on possible interventions. This study attempts to fill this knowledge gap.

## MATERIALS AND METHODS

### Study design

An analytical cross-sectional study was conducted in August 2011.

### Sample-size

The sample-size was based on the 30×30 cluster methodology. This sample-size ensures that the estimated prevalence will be within plus or minus 5% of the true prevalence with a probability of 95%, irrespective of the value of population proportion of the variable of interest, and assuming a correction factor of 2 (the ‘design effect’) for cluster sampling.

For the purpose of correcting for any spoiled or damaged questionnaire, an extra 10% mothers were sampled bringing the actual number of mothers to 991.

### Study population and sampling

The study population comprised mothers of children aged 0-36 month(s), who sought CWC services in the 12 months before the commencement of the study.

The primary sampling units (PSUs) were communities. The clusters were selected based on the 30×30 two-stage cluster-sampling procedure from across three regions (Upper West, Upper East, and Northern) constituting Northern Ghana. The sample clusters were selected using probability-proportional-to- size method. In each cluster, systematic random sampling was used in selecting each participating household. All the households in each cluster were serially numbered; the total number of households in a cluster was divided over the sample-size of 33 to give the sampling interval. The first household was randomly selected by picking any number within the sample interval. Subsequent selections were made by adding the sampling interval to the selected number. This was done until the desired sample-size was obtained. The eligible respondents included all children aged 0-36 month(s) and their mothers. In each household, only one eligible respondent was randomly selected for interview, and anthropometric assessment of her child was done.

### Variables and measurements

The main dependent variable was stunting as measured by height-for-age z-scores (HAZ). The height-for-age z-score was generated using the World Health Organization (WHO) 2006 growth standards. The variable was coded as 1 (stunted) if HAZ was <−2, and 0 (not stunted) if HAZ was ≥−2. Maternal childcare knowledge was the main predictor variable. The covariates included: child's characteristics, maternal demographic characteristics, mother's educational level, and household wealth index.

Demographic and feeding practices were assessed through using a structured questionnaire administered to mothers/caregivers. Trained fieldworkers assisted with the completion of maternal childcare nutritional knowledge questionnaire (CNKQ) through one-on-one interviews with the respondents. The maternal attributes of age, ethnicity, level of attainment of formal education, marital status, and type of occupation were recorded.

### Childcare nutritional knowledge of mothers

A CNKQ was specifically developed for the purpose of this study. The aspects of maternal childcare nutritional knowledge studied included: (i) age for introducing semi-solid foods into a child's diet; (ii) mother's knowledge of importance of feeding colostrum to the child; (iii) giving fluids during diarrhoea; (iv) giving semi-solid foods during diarrhoea; and (v) correct preparation of oral rehydration solution (ORS). Based on the responses, a score of 1 was assigned for each valid answer, with a maximum possible score of 5. For example, giving less fluids or withholding semi-solid foods for a child having diarrhoea attracted a score of −1. Giving the same amounts as before the diarrhoeal episode scored zero. Giving more fluids and semi-solids scored 1. A mother's overall knowledge of recommended childcare practices was rated by calculating the total of all the valid responses she made. The overall composite childcare knowledge index, therefore, ranged from a minimum of −2 to a maximum of 5, and terciles were created. Women who scored below the sample mean score were classified as having low childcare nutritional knowledge, and women with a score of at least the sample mean score were classified as having high score. This was necessitated because we needed to meet certain criteria for running some statistical analysis (e.g. chi-square). With more than two categories, we realized we could not satisfy the criteria for chi-square analysis (that is, each cell should contain at least five units).

### Determination of educational level

The educational level was based on the highest level attained according to the Ghanaian System where primary education consists of six years of formal education; Junior High School (JHS) is of nine years; and Senior High School (SHS) is of 13 years. An individual with tertiary-level education spends at least 17 years acquiring formal education. This variable has three categories: no education, primary or junior secondary, and senior secondary education or higher.

### Determination of household wealth index

A household wealth index based on household assets and housing quality was used as a proxy indicator for socioeconomic status (SES) of households. An absolute measure of household wealth (wealth index) used in this study was based on an earlier concept developed by Garenne and Hohmann (11) determined by the sum of dummy variables created from information collected on housing quality (floor, walls, and roof material), availability of water and type of toilet facility, and ownership of household durable goods and livestock (e.g. bicycle, television, radio, motorcycle, sewing machine, telephone, car, refrigerator, mattress, and bed). These facilities or durable goods are often regarded as modern goods that have been shown to reflect household wealth. A household with zero index score means that the household had not a single modern good. The scores were, thus, added up to give the proxy household wealth index. The index varied from 0 to 18. Terciles were created but the middle category had no adequate numbers to permit for some analyses (e.g. chi-square requires a minimum of 5 in each cell to be valid). The middle category was, thus, collapsed with the highest tercile.

The main aim of creating the index was to categorize households into SES groupings to compare the difference in the prevalence of child growth outcome between the groups of the lowest and the highest SES.

### Assessment of nutritional status

Nutritional status was measured in terms of height-for-age z-scores (HAZ) and weight-for-height z-scores (WHZ). Anthropometric variables used in the study were weight and height. The details of measurement are described below:

*Age*: The exact age of the child was recorded in months based on information contained in booklets on child health records, birth certificates, and baptismal cards.
*Weight*: The weight of children was assessed with Seca Electronic UNISCALE (SECA 890). Weight was measured to the nearest gramme. The batteries were replaced after every cluster (minimum of 80 readings). This was done to ensure that weak batteries do not affect the readings on the scales.
*Height*: Recumbent length was measured with a specialized wooden device (i.e. an infantometer). The length-board had the precision of 0.1 cm. The length of children aged less than two years (i.e. up to and including 23 months) was measured in a lying position. The child was placed on his/her back between the slanting sides. The head was placed in a position so that it is against the top end. The knees were gently pushed down by a helper. The foot-piece was then moved towards the child until it presses softly against the soles of the child's feet and the feet are at right angles to the legs. For children aged 24-36 months, stature was measured in a standing position.
*Bilateral oedema*: This was diagnosed by placing both thumbs on the upper side of the feet and applying pressure for about three seconds. Oedema was considered to be present if a skin depression remained after the pressure was released.

### Data processing and analysis

The data collected for the study was analyzed using the Predictive Analytical Software (PASW) formerly known as Statistical Package for Social Sciences (SPSS) (version 18).

The Emergency Nutrition Assessment (ENA) for SMART software (2012 version) was used for the anthropometric data analysis. The WHO 2006 growth reference values were used as the standard for calculating weight-for-age z-score (WAZ), height-for-age z-score (HAZ), and weight-for-height z-score (WHZ). Before performing the anthropometric calculations for WAZ, HAZ, and WHZ, the data were cleaned to remove the outliers as defined by Emergency Nutrition Assessment (ENA) software (2011 version). Outliers identified as likely errors were weight-for-height z-scores above +6 or below −4, weight-for-age z-scores above +6 or below −6, height-for-age z-scores above +6 or below −6. Consequently, 10 outliers were detected and removed.

Descriptive and multivariate analyses were conducted to identify key predictors associated with growth of children. For bivariate analyses, chi-square test was used for studying the significance of difference between proportions while Analysis of Variance (ANOVA) was used for testing group difference of quantitative variables. Analysis of Covariance (ANCOVA) was done to study the effect of the mothers’ nutritional knowledge on the children's nutritional status, controlling for potential confounders, including sociodemographic variables and maternal educational levels.

Multivariate regression analysis was used in assessing the association between childcare knowledge index and nutritional status (i.e. WAZ, HAZ, and WHZ), controlling for extraneous variables found to be significant in the bivariate analyses. Statistical difference was considered significant if the p value was <0.05, and 95% confidence intervals (CI) were calculated for all main outcome measures that met the normality and homogeneity assumption criteria.

To investigate interactions between maternal childcare nutritional knowledge score and household wealth index, these quantitative variables were converted into binary categorical variables. For example, household wealth index was used as proxy measure of socioeconomic status (SES) of household members. This was done by splitting the sample into low/high SES based on the distribution of variable in the sample. Essentially, interaction means a particular explanatory variable [in this case, maternal childcare nutritional knowledge was significant determinant of an outcome (e.g. mean height-for-age z-score)] in subgroup of children of high socioeconomic status but not among children of lower socioeconomic status. The presence of interaction was verified from the significance level of the standardized beta coefficients obtained in the multiple regression analyses of the subgroup.

### Ethical clearance

Ethical clearance was sought and obtained from the School of Medicine and Health Sciences (SMHS) Ethics Committee, University for Development Studies. Permission for the study was obtained from authorities of the Ghana Health Service. Each study participant, after being briefed and offered the opportunity to ask questions about the study, was provided with individual informed written consent form to sign or thumbprint.

## RESULTS

### Characteristics of the sample

Table 1 presents the characteristics of the sample. The mean age of children covered in the study was 16.6±10.5 months. There were slightly more boys than girls. In terms of education, majority of mothers interviewed [788 (79.5%)] had no formal education.

### Maternal knowledge level in childcare practices

Out of the 991 mothers, 676 (68.2%) were classified as having low knowledge with regard to appropriate childcare practices while 315 mothers (31.8%) were having high knowledge levels in childcare practices. The mean score of mothers’ nutritional knowledge on childcare practices was 2.0±1.5 out of a maximum of 5. Mothers of children aged less than 6 months scored the lowest childcare knowledge index. Knowledge about the nutritional management of diarrhoea was particularly low. Less than 20.0% of mothers knew that giving more semi-solid foods to the child during diarrhoea was a recommended childcare practice (Table 2).

**Table 1.** Characteristics of samples (N=991)

Characteristics	Mean	Standard deviation
Child's characteristics		
Age (months)	16.6	10.5
Male-female ratio	1.3	−
Mean weight (kg)	9.0	2.5
Mean height (cm)	74.9	10.5
Mother's characteristics		
Educational level of mother	No.	%
None	788	79.5
Primary	108	10.9
JSS/Middle	63	6.4
Secondary	26	2.6
Tertiary	6	0.6
Marital status		
Single	35	3.5
Married	939	94.8
Widowed	12	1.2
Divorced	5	0.5

**Table 2.** Maternal knowledge levels in childcare practices (N=991)

Variable	No.	%
Proportion of mothers knowing the importance of feeding colostrum to the child	825	83.2
Proportion of mothers knowing the age for introducing semi-solid foods into a child's diet	438	44.2
Proportion of mothers giving more fluids to the child during diarrhoea	227	22.9
Proportion of mothers giving more semi-solid foods to the child during diarrhoea	189	19.1
Proportion of mothers who described the correct preparation of oral rehydration solution (ORS)	610	61.6
Proportion of mothers scoring a high childcare knowledge index	315	31.8

### Nutritional status of children

Of the 991 children, 25.1% were stunted (HAZ <−2) while 13.7% were suffering from global acute malnutrition (WHZ <−2) (Table 3).

### Relationship between maternal childcare knowledge level and childhood anthropometric indices

There was no discernible association between maternal childcare knowledge scale and child's nutritional indicators among children below six months. However, among children aged 6-36 months, there was significant association between nutritional status and maternal childcare knowledge. Analysis of Variance (ANOVA) showed that the unadjusted mean WAZ, WHZ, and HAZ in children born to mothers having higher levels of maternal childcare knowledge were significantly higher than of those in children of mothers who had lower knowledge levels in childcare practices (Table 4a).

In Analysis of Covariance (ANCOVA), while adjusting for age of the child, gender of child, maternal educational level, and the household wealth index, the mean HAZ was 0.44 significantly higher (CI 0.03-0.85, p=0.03) among children born to mothers of higher childcare nutritional knowledge compared to children born to women with knowledge of lower tercile. There was no significant difference in mean HAZ between lower and middle terciles of maternal childcare knowledge. Similarly, adjusting for age of the child and maternal educational level, difference in WAZ between children born to women of high childcare nutritional knowledge level and children born to women of lower nutritional knowledge in caring practices was 0.45 (CI 0.09-0.80, p=0.008). However, maternal childcare knowledge index was not an important determinant of mean WHZ in this multivariate analysis. Table 4b shows the pair-wise comparisons for dependent variable of mean height-for-age z-score.

**Table 3.** Prevalence of underweight, wasting, and stunting among children aged 0-36 month(s)

Parameter	WAZ	WHZ	HAZ
Mean	−0.94	−0.48	−1.05
SD	±1.4	±1.48	±1.57
% below −2 SD	20.1 (199)*	13.7 (136)*	25.1 (249)*
No. examined (N)	991	991	991

*Numbers in brackets are absolute numbers

**Table 4a.** Distribution of anthropometric indicators among children aged 6-36 months according to maternal childcare knowledge

Maternal knowledge level		n	Mean	Standard deviation	95% confidence interval for mean		Test statistic
					Lower bound	Upper bound	
WAZ	Lower tercile	302	−1.24	1.33	−1.39	−1.08	F (2,809)=8.0, p<0.001
	Middle tercile	401	−1.21	1.31	−1.34	−1.09	
	Higher tercile	107	−0.68	1.15	−0.91	−0.46	
	Total	810	−1.15	1.31	−1.24	−1.06	
WHZ	Lower tercile	302	−0.55	1.53	−0.72	−0.37	F (2,809)=4.8, p=0.009
	Middle tercile	401	−0.65	1.42	−0.79	−0.52	
	Higher tercile	107	−0.16	1.49	−0.45	0.12	
	Total	810	−0.55	1.48	−0.65	−0.45	
HAZ	Lower tercile	302	−1.46	1.62	−1.64	−1.27	F (2,809)=3.8, p=0.022
	Middle tercile	401	−1.22	1.42	−1.36	−1.08	
	Higher tercile	107	−1.02	1.72	−1.35	−0.69	
	Total	810	−1.28	1.54	−1.39	−1.18	

### Relationship between maternal educational level and childcare knowledge of the mother

Mothers of higher educational standing demonstrated more possession of knowledge in childcare compared to mothers of low educational level [(3.0±2.0 vs 1.8±1.4), F (2,990)=15.4, p<0.001]. Similarly, a higher proportion of women who attained higher educational level possessed high knowledge levels (chi-square=62.0, p<0.001) (Table 5).

However, socioeconomic status of the mother was not a determinant of mother's knowledge level of recommended childcare practices.

### Maternal knowledge in childcare practices and its relationship with child growth

A composite childcare knowledge index was developed, and its relationship with child growth was investigated. Multivariate analysis showed that, after adjusting for potential confounders, there was a significant positive association between the childcare knowledge index and mean HAZ (β=0.10, p=0.005) but was not associated with mean WHZ.

Using ANCOVA, after adjusting for educational level of the mother and gender of the child, there was a significant interaction between maternal childcare knowledge and age of the child on mean HAZ [F (1,990)=78.2, p<0.001] and household wealth index on mean HAZ [F (1,990)=16.7, p<0.001]. In view of the strong interaction between socioeconomic status and level of maternal knowledge, the association between level of maternal nutritional knowledge and child's nutritional status were tested using multivariate regressions within each socioeconomic subgroup.

### Determinants of HAZ among children aged 6-36 months

Maternal childcare knowledge interacted strongly not only with socioeconomic status of households but also with age of the child. Consequently, there was no discernible association between maternal childcare knowledge index and child's nutritional indicators among children below six months. However, among children aged 6-36 months, there was a significant association between mean HAZ and maternal childcare knowledge but not with WHZ in multivariate regression analyses. Table 6 shows the bivariate analysis of predictors of chronic malnutrition among children aged 0-36 month(s).

Modelling was done using multiple regression analysis (stepwise method). Maternal knowledge level variable was introduced first into the model, followed by individual potential confounders that included the socioeconomic status of mother. The significant independent determinants of mean HAZ were age of the child, socioeconomic status of the household, educational level of the mother, knowledge level of the mother in recommended childcare practices, and gender of the child. Female children were generally taller (β=0.07, p=0.04), and a unit increase of mother's knowledge in childcare practices was associated with an increase in HAZ by 0.10 standard units. The multivariate analysis also showed that, after adjusting for potential confounders, there was a significant positive association between the childcare knowledge index and mean HAZ (β=0.10, p=0.005) (Table 7).

**Table 4b.** Pair-wise comparisons for dependent variable of mean height-for-age z-score

(I) Terciles for knowledge score	(J) Terciles for knowledge score	Mean difference (I-J)	Significance^a^	95% confidence interval for difference^a^	
				Lower bound	Upper bound
Lower tercile	Middle tercile	−0.24	0.107	−0.50	0.03
	Higher tercile	−0.44*	0.029	−0.85	−0.03
Middle tercile	Lower tercile	0.24	0.107	−0.03	0.50
	Higher tercile	−0.20	0.513	−0.60	0.19
Higher tercile	Lower tercile	0.44*	0.029	0.03	0.85
	Middle tercile	0.20	0.513	−0.19	0.60

Based on estimated marginal means;

^a^Adjustment for multiple comparisons: Sidak;

*Mean difference is significant at the 0.05 level

**Table 5.** Relationship between maternal educational level and childcare knowledge of the mother

Classification of maternal educational level	Count	Tercile for knowledge score			Total
	%	Lower tercile	Middle tercile	Higher tercile	
Nil	Count	322	401	65	788
	%	40.9	50.9	8.2	100.0
Low	Count	55	90	26	171
	%	32.2	52.6	15.2	100.0
High	Count	8	8	16	32
	%	25.0	25.0	50.0	100.0
Total	Count	385	499	107	991
	%	38.8	50.4	10.8	100.0

### Effect of the knowledge level of the mother on HAZ in wealthy families

The effect of maternal childcare nutritional knowledge level on chronic malnutrition among children aged 6-36 months was more significant among children from households of high socioeconomic status but maternal educational level was not (Table 8).

**Table 6.** Bivariate analysis of predictors of chronic malnutrition among children aged 0-36 month(s)

Characteristics	No.	Classification of chronic malnutrition		
		Normal	Stunted	Test statistic
		n (%)	n (%)	
Age of child (in completed months)				
Less than 6 months	181	171 (94.5)	10 (5.5)	χ=53.3, p<0.001
6-11	192	145 (75.5)	47 (24.5)	
12-23	321	233 (72.6)	88 (27.4)	
24-36	297	193 (65.0)	104 (35.0)	
Total	991	742 (74.9)	249 (25.1)	
Household wealth index of woman				
Low	662	472 (71.3)	190 (28.7)	χ*^2^*=3.6, p<0.001
High	329	270 (82.1)	59 (17.9)	
Total	991	742 (74.9)	249 (25.1)	
Maternal childcare knowledge				
Low tercile	385	275 (71.4)	110 (28.6)	χ*^2^*=4.3, p=0.1
Middle tercile	499	387 (77.6)	112 (22.4)	
High tercile	107	80 (74.8)	27 (25.2)	
Total	991	742 (74.9)	249 (25.1)	
Gender of baby				
Male	554	402 (72.6)	152 (27.4)	χ*^2^*=3.6, p=0.06
Female	437	340 (77.8)	97 (22.2)	
Total	991	742 (74.9)	249 (25.1)	

**Table 7.** Determinants of HAZ (whole sample)

Parameter	Standardized coefficient (beta)	Significance	95% confidence interval for β	
			Lower bound	Upper bound
(Constant)	−	0.001	−2.24	−1.21
Age of child in months	−0.23	0.001	−0.05	−0.03
Maternal childcare knowledge index	0.10	0.005	0.03	0.16
Household wealth index of mother	0.15	0.001	0.27	0.70
Educational level of woman	0.09	0.008	0.07	0.48
Gender of child	0.07	0.04	0.01	0.42

**Table 8.** Determinants of HAZ (households with high socioeconomic status)

Parameter	Standardized coefficient (beta)	Significance	95% confidence interval for β	
			Lower bound	Upper bound
(Constant)	−	0.15	−1.23	0.19
Age of child in months	−0.32	0.001	−0.08	−0.04
Maternal childcare knowledge index	0.15	0.014	0.03	0.28
Educational level of woman	0.06	0.32	−0.15	0.46
Gender of child	0.06	0.27	−0.16	0.57

### Effect of the knowledge level of the mother on HAZ in poor families

Among women of low socioeconomic status, maternal childcare knowledge index was an insignificant determinant of mean HAZ. Rather, the educational level was a significant determinant of mean HAZ (Table 9).

**Table 9.** Determinants of HAZ (households with high socioeconomic status)

Parameter	Standardized coefficient (beta)	Significance	95% confidence interval for β	
			Lower bound	Upper bound
(Constant)	−	0.001	−1.88	−0.884
Age of child in months	−0.19	0.001	−0.04	−0.02
Maternal childcare knowledge index	0.07	0.09	−0.01	0.15
Educational level of woman	0.12	0.006	0.12	0.67
Gender of child	0.07	0.08	−0.03	0.47

## DISCUSSION

In this study, we investigated the effect of maternal childcare knowledge on the nutritional status of children, controlling for potential confounders, including mothers’ educational level, age of the child, and household wealth index. We also determined whether literate mothers had better childcare knowledge.

The major finding of our study was that maternal childcare knowledge index was strongly and positively associated with child growth indices, especially mean HAZ. The other key finding from the analysis was that maternal childcare knowledge was an important determinant of child growth among children aged 6-36 months, especially children from households with high socioeconomic status but not among children below six months.

The association between the nutritional status of the child and childcare knowledge index of the mother was, however, modified by socioeconomic status. Therefore, the level of maternal knowledge in recommended childcare practices impacts on child's nutritional status but this is dependent on the socioeconomic status of the household.

### Prevalence of malnutrition

The prevalence of stunting and wasting in the study area was comparable to the prevalence in the 2008 Ghana Demography and Health Survey reported for the Northern Region. The prevalence of chronic malnutrition (HAZ <−2 z-scores) in the study area could be described as serious according to WHO classification for assessing severity of malnutrition by prevalence range among under-five children (12). Global acute malnutrition (GAM), on the other hand, according to this classification, was also serious.

### Maternal knowledge level in childcare practices

Mothers of children aged less than 6 months scored the lowest childcare knowledge index, and this may be explained by the fact that such mothers may be less exposed to the caring practices that were measured in this study. Children aged less than 6 months may fall ill less often compared to their older counterparts, and their mothers will be less conversant with caring practices associated with illnesses, such as diarrhoea.

Generally, knowledge about the nutritional management of diarrhoea was particularly low. Less than 20.0% of mothers knew that giving more semi-solid foods to the child during diarrhoea was a recommended childcare practice.

Both literate and non-literate mothers possessed some levels of childcare nutritional knowledge, although mothers of higher educational standing demonstrated more possession of knowledge in childcare compared to mothers of low educational level. Non-literate mothers usually obtain nutrition information from antenatal and postnatal care whereas literate ones may obtain such information from other sources, including reading literature.

### Maternal knowledge in childcare practices and its relationship with child growth

A composite childcare knowledge index was developed, and its relationship with child growth was investigated. Evidence from the analyses of data showed that the main predictor variable (maternal childcare knowledge) interacted strongly not only with socioeconomic status of households but also with age of the child. Consequently, there was no discernible association between maternal childcare knowledge scale and child's nutritional indicators among children below six months, perhaps due to the fact that the prevalence of undernutrition was low in this age-group, and most of the caring practices measured really did not apply to them. However, among children aged 6-36 months, there was a significant association between mean HAZ and maternal childcare knowledge but not with WHZ in multivariate regression analyses. Whereas HAZ measures chronic undernutrition, WHZ measures acute undernutrition, and the determinants of these differ. As seen in this study, maternal childcare knowledge was not an important predictor of acute undernutrition. Application of maternal care behaviours through acquired knowledge may not be responsive to short-term single events, such as improved weight gain but might have a cumulative effect on long-term growth.

Multivariate analysis showed that, after adjusting for potential confounders, there was a significant positive association between the childcare knowledge index and mean HAZ but was not associated with mean WHZ. The strength of association increased among women of high socioeconomic status and who had children aged 6-36 months. There was, however, no significant association among women of low socioeconomic status. The impact of maternal care knowledge was particularly stronger among children who were aged more than six months. The results closely agree with the findings of previous studies in India where mothers’ nutritional knowledge was positively associated with improved growth of children aged 0-36 month(s) but did not affect the weight-for-age and height-for-age of the children aged 37-72 months (13). In the present study, childcare knowledge index did not associate with mean WHZ but, in the Indian study, acute malnutrition, as measured by weight-for-height, was significantly related to the mothers’ knowledge in children of all age-groups. In rural Haiti, mothers’ nutritional knowledge scores were associated with the long-term wellbeing of children represented by height-for-age (6).

Findings from an earlier study conducted in Accra, Ghana, suggested that knowledge gained through informal education could mitigate the negative effects of poverty and low maternal schooling on children's height-for-age z-score (HAZ) (14) while others concluded that, among the poorest, nutritional knowledge appeared to be insufficient for mothers to be able to adopt optimal childcare practices that may improve child's nutritional status (15).

Evidence from our data suggests that adequate knowledge of childcare practices did not make any significant impact on child growth in poor households. This may be due to the fact that adequate care requires more than knowledge. Control of resources is another essential ingredient for care, and this was amply demonstrated by our data. The findings of the earlier study by Ruel *et al*. (1999) in Southern Ghana are directly opposite to our findings obtained from Northern Ghana. A possible explanation could be that we used knowledge of care variable whereas the Southern Ghana study used actual care practice variable. The two variables are not the same but are related. It is important to note that the same authors had opposite findings in Lesotho where care practices had a greater impact on children's nutrition among poorer households and made no difference among the upper income tercile. The authors explained that differences in absolute levels of poverty between Accra and rural Lesotho were probably responsible for these contrasting results. Obviously, there were marked socioeconomic disparities between Southern and Northern Ghana because Northern Ghana had the highest poverty levels. The three regions of Northern Ghana were persistently the poorest regions and where economic growth had been difficult to stimulate. This probably accounts for the inconsistencies between our study and the Southern Ghana study. The big lesson from these studies emphasizes the importance of designing context-specific nutrition interventions based on empirical evidence.

In another study carried out in the Volta Region of Ghana, maternal nutritional knowledge was independently associated with nutritional status but maternal education, on the other hand, was not found to be independently associated with nutritional status (16). Some other studies reported a positive relationship between maternal nutritional knowledge and child's nutritional status represented by height-for-age ((5,6) but others have shown the contrary (8,9,17). In studies that found no association between maternal nutritional knowledge and child's nutritional status, the role of socioeconomic disparities of the households was not investigated.

It is a common belief that most of the nutritional behaviours necessary for improved nutritional status need little economic investment to succeed and yet malnutrition continues to be a major public-health problem in developing countries. The results of this study suggest that mothers of low socioeconomic status, living in adverse conditions with inadequate resources, are unable to apply acquired nutritional knowledge successfully in childcare to benefit their children.

### Strengths and limitations

The cross-sectional nature of the study limits our ability to draw any causal conclusions since the problem of endogeneity cannot be ruled out. The main independent variable (maternal childcare knowledge) might be correlated with the error term in the regression, leading to biased estimates. Recall bias was also possible and might affect the validity of the findings. Despite these limitations, our results have shed more light on the association between maternal childcare knowledge and the nutritional status of children aged 6 to 36 months in developing-country settings.

### Conclusions and recommendations

Our data provided evidence that maternal childcare knowledge index of mothers was strongly and positively associated with mean HAZ of children. The association between the nutritional status of the child and childcare knowledge index of the mother was, however, modified by socioeconomic status and age of the child. The results suggest that mothers of low socioeconomic status, living in adverse conditions with inadequate resources, may be unable to apply acquired nutritional knowledge successfully in childcare to benefit their children.

Increase in maternal childcare knowledge through nutrition/health education may contribute significantly to child's nutritional status in Ghana if there is concurrent improvement in socioeconomic circumstances of women living in deprived rural communities.

### What this study has added

This study is the first to show that maternal childcare nutritional knowledge has independent effect on child growth in rural Northern Ghana where resource constraints are very prominent. Our findings are in contrast with an earlier study that was conducted in urban area of Southern Ghana, which advocated that better childcare practices are maximized in poor households. The policy implication from the findings of the present study emphasizes the importance of designing context-specific nutrition interventions based on empirical evidence.
